# Cannabinoids Alleviate Experimentally Induced Intestinal Inflammation by Acting at Central and Peripheral Receptors

**DOI:** 10.1371/journal.pone.0109115

**Published:** 2014-10-02

**Authors:** Jakub Fichna, Misha Bawa, Ganesh A. Thakur, Ritesh Tichkule, Alexandros Makriyannis, Donna-Marie McCafferty, Keith A. Sharkey, Martin Storr

**Affiliations:** 1 Snyder Institute for Chronic Disease, Department of Medicine, University of Calgary, Calgary, Alberta, Canada; 2 Department of Physiology and Pharmacology, University of Calgary, Calgary, Alberta, Canada; 3 Hotchkiss Brain Institute, University of Calgary, Calgary, Alberta, Canada; 4 Department of Biochemistry, Medical University of Lodz, Lodz, Poland; 5 Center for Drug Discovery, Department of Pharmaceutical Sciences, Northeastern University, Boston, Massachusetts, United States of America; 6 Division of Gastroenterology, Department of Medicine, University of Munich, Munich, Germany; University of South Carolina School of Medicine, United States of America

## Abstract

**Background and Aims:**

In an attempt to further investigate the role of cannabinoid (CB) system in the pathogenesis of inflammatory bowel diseases, we employed two recently developed ligands, AM841 (a covalently acting CB agonist) and CB13 (a peripherally-restricted CB agonist) to establish whether central and peripheral CB sites are involved in the anti-inflammatory action in the intestine.

**Methods and Results:**

AM841 (0.01, 0.1 and 1 mg/kg, i.p.) significantly decreased inflammation scores in dextran sulfate sodium (DSS)- and 2,4,6-trinitrobenzene sulfonic acid (TNBS)-treated mice when administered before induction of colitis or as a treatment of existing intestinal inflammation. The effect was absent in CB_1_, CB_2_ and CB_1/2_-deficient mice. A peripherally-restricted agonist CB13 did not alleviate colitis when given i.p. (0.1 mg/kg), but significantly decreased inflammation score after central administration (0.1 µg/animal).

**Conclusions:**

This is the first evidence that central and peripheral CB receptors are responsible for the protective and therapeutic action of cannabinoids in mouse models of colitis. Our observations provide new insight to CB pharmacology and validate the use of novel ligands AM841 and CB13 as potent tools in CB-related research.

## Introduction

Ulcerative colitis (UC) and Crohn's disease (CD) are two major types of inflammatory bowel diseases (IBD). In clinical terms, UC is limited to an inflammation of the mucosal lining of the colon and rectum, while CD is a transmural inflammation of the intestinal wall that can occur throughout the length of the gastrointestinal (GI) tract (for review see: [1], [2]). The aetiology of IBD remains unclear, but epidemiologic and molecular studies suggest that genetic factors, a breakdown in mucosal defense, environmental triggers (notably the luminal microbiota) and altered immune responses within the GI tract converge to produce chronic inflammation. Both UC and CD have elements of the innate and acquired immune responses that underlie the inflammation in the gut. In addition, there is an important involvement of the nervous system in the pathophysiology of colitis, including neuronal signalling in the enteric nervous system (ENS) and extrinsic autonomic and primary afferent pathways involving the central nervous system (CNS) [3]–[5]. One important neural signaling system implicated in the pathophysiology of colitis is the endogenous cannabinoid system (ECS).

The ECS consists of cannabinoid (CB) receptors, their endogenous ligands, and the enzymes involved in endocannabinoid synthesis, transport and inactivation (for review see: [6]). To date, two CB receptors, CB_1_ and CB_2_, have been identified in mammalian tissues. The CB_1_ receptors are localized principally in the CNS [7], [8], but are also found in the peripheral tissues, like ENS, small intestine, spleen and leukocytes [9]–[12]. The CB_2_ receptors are mainly distributed in immune cells, including B and T lymphocytes, macrophages and neutrophils [10], [13]. However, the presence of CB_2_ in the ENS has also been detected [14]. The endogenous ligands of CB receptors, *N*-arachidonoylethanolamine (anandamide) and 2-arachidonoyl glycerol (2-AG) are found in the mammalian digestive tract, where they play an important role in the regulation of GI motility, secretion, proliferation, and immune functions (for review see: [6]).

Recently, it has been shown that CB receptor activation prevents and ameliorates symptoms of inflammation in animal models of experimental colitis [15]–[17] and the potential use of therapeutics interacting with ECS has attracted attention as a promising strategy for the treatment of IBD in humans. The molecular mechanisms of the anti-inflammatory actions of cannabinoids include CB_1_-promoted epithelial wound healing [18], CB_2_-mediated inhibition of cytokine and chemokine release, and immune cell recruitment [19], [20], as well as CB-mediated reduction of motility in inflamed intestine [21], [22]. It was also observed that the blockade of the CB_1_ receptors by antagonists or genetic manipulations increased the severity of colitis [15]. The increase of CB_2_ expression in the colonic epithelium of IBD patients, which plays a key role in host defence, translated well the key findings from animal tissues to humans [18], [19].

A better understanding of mechanisms that protect against inflammation is required for the development of novel, efficient therapeutic strategies for the treatment of IBD. Although multiple lines of evidence clearly indicate the anti-inflammatory effect of cannabinoids on colitis, several questions remain unanswered. In the present study we used two recently developed cannabinergic ligands, a covalently acting CB agonist AM841 [23] and a peripherally-restricted CB agonist CB13 [24] to determine whether both, central and/or peripheral receptors mediate the anti-inflammatory actions of cannabinoids. In addition, we wanted to confirm whether cannabinoids have the potential to protect against and to treat colitis at an advanced stage of its development. To examine the anti-inflammatory actions of cannabinergic ligands, we used two well-established animal models of colitis, induced by 2,4,6-trinitrobenzenesulfonic acid (TNBS) and dextran sulfate sodium (DSS).

## Materials and Methods

### Animals

Male CD1 mice, and CB receptor knockout (CB_1_^-/-^, CB_2_^-/-^, CB_1/2_^-/-^) mice and their littermates on a C57Bl/6 background, weighing 25–30 g, were used. Unless otherwise stated, experiments were performed on CD1 mice (Charles River, Canada). The animals were housed at a constant temperature (22°C) and maintained under a 12-h light/dark cycle in sawdust-lined plastic cages with free access to laboratory chow and tap water. The CB knockout mice were bred in our facility at the University of Calgary, Canada. All CB knockout mice were genotyped using an established protocol [19] and confirmed as homozygous CB receptor gene-deficient animals prior to inclusion in the study.

All animal protocols were approved by the University of Calgary Animal Care Committee (protocol number M07102), and the experiments were performed in accordance with the guidelines of the Canadian Council on Animal Care.

### Induction of colitis

DSS colitis. Colitis was induced by the addition of dextran sulfate sodium (DSS) to drinking water (days 0–5; 4% wt/vol; molecular weight, 36,000–50,000; MP Biomedicals, Aurora, OH, USA, Lot No. 5237K) [25]. On days 6 and 7 the animals received water without DSS.

TNBS colitis. Colitis was induced by intracolonic (i.c.) administration of 2,4,6-trinitrobenzene sulfonic acid (TNBS), as described previously [25].

### Pharmacological treatments

Drugs were administered intraperitoneally (i.p.) or centrally (i.c.v.). AM841 (0.01–1 mg/kg, once or twice daily) and CB 13 (0.1 mg/kg, i.p. and 0.1 µg/animal, i.c.v., once daily) were dissolved in vehicle (2% dimethyl sulfoxide and 1% Tween 80 in saline) and injected 15 min before and then for 3 (TNBS model) or 7 days (DSS model) after the induction of colitis (n = 6–8 animals per treatment group). Control animals received vehicle alone. To investigate whether there is a beneficial effect of AM841 on established colitis, the drug was injected once or twice daily from day 4 following induction of DSS colitis. The doses of AM841 and CB 13 were established based on preliminary experiments.

For the chemotactic assays, a stock solution of AM841 in DMSO (10^−2^ M) was prepared and diluted accordingly.

### Evaluation of colonic damage

DSS colitis. Mice were sacrificed by cervical dislocation on day 7 following addition of DSS to the drinking water. The entire colon was immediately dissected and weighed with fecal content. Colon was then opened along the mesenteric border and fecal material removed by gently rinsing with PBS. A total macroscopic damage score was calculated for each animal as described previously [25].

TNBS colitis. Animals were sacrificed by cervical dislocation 3 days after TNBS infusion. The colon was quickly removed, opened longitudinally, washed with phosphate buffered saline (PBS), and immediately examined. Macroscopic colonic damage was assessed by an established semiquantitative scoring system by adding individual scores for ulcer, colonic shortening, wall thickness, and presence of hemorrhage, fecal blood, and diarrhea, as described previously [25].

### Determination of Tissue Myeloperoxidase Activity

Myeloperoxidase (MPO) activity was measured to assess the degree of granulocyte infiltration. Colon segments (1 cm) were weighed and processed immediately after isolation, according to the method described previously [25]. MPO was expressed in milliunits per gram of wet tissue, 1 unit being the quantity of enzyme able to convert 1 µmol of hydrogen peroxide to water in 1 min at room temperature. Units of MPO activity per 1 min were calculated from a standard curve using purified peroxidase enzyme.

### Histology

Following macroscopic scoring, segments of the distal colon were stapled flat, mucosal side up, onto cardboard and fixed in 10% neutral-buffered formalin for 24 h at 4°C. Specimens were then dehydrated, embedded in paraffin, sectioned at 5 µm and mounted onto slides. Sections were stained with hematoxylin and eosin and examined using a Zeiss Axioplan microscope (Carl Zeiss, Toronto, ON, Canada). Photographs were taken using a digital imaging system consisting of a digital camera (Sensys, Photometrics, Tucson, AZ, USA) and image analysis software (V for Windows, Digital, Optics, Auckland, New Zealand). A microscopic total damage score was assessed as described previously [25].

### Isolation of bone marrow-derived neutrophils and chemotactic assay

Neutrophils were isolated from untreated CD1 mice as described previously [26]. They were pre-incubated for 30 min with AM841 (0.1–10 nM) or RPMI alone to determine the baseline migration. After pre-incubation, 0.2 ml of cell suspension was transferred into the upper well of a 6.5 mm insert in 24-well Transwell chemotaxis chamber (Corning Life Sciences, Lowell, MA, USA) with an 8-µm polycarbonate membrane. Bottom wells of the plate were filled with 0.6 ml of a 10 nM solution of the synthetic neutrophil chemoattractant N-formyl-peptide (fMLP) (Phoenix Pharmaceuticals, Burlingame, CA, USA). Plates were incubated at 37°C in a humidified incubator for 3 h. Cells which migrated to the lower chamber were counted in a hemocytometer.

### Splenocyte cell isolation and differentiation into macrophages

Spleens, isolated from untreated CD1 mice, were placed into separate sterile Falcon tubes containing RPMI medium supplemented with 10% FBS (CanSera, Toronto, ON, Canada), 2% penicillin/streptomycin and 25 mM 4-(2-hydroxyethyl)-1-piperazineethanesulfonic acid (HEPES) (both from Invitrogen Life Technologies, Burlington, ON, Canada) and then mechanically disrupted by pressing through a Falcon cell strainers (100 µm pore size, BD Biosciences, Mississauga, ON, Canada). Cells were centrifuged at 1400 rpm for 5 min and the pellets resuspended in 2 ml of ACK lysis buffer (150 mM NH_4_Cl, 10 mM KHCO_3_, and 0.1 mM EDTA) for 90 s to remove erythrocytes. Following centrifugation, cells were resuspended in medium at 5×10^6^/ml for immediate use.

For differentiation into a macrophage-like phenotype, splenic cells were transferred into 24-well plates (1 ml/well), incubated for 2 h at 37°C under 5% CO_2_ and washed twice with PBS to remove non-adherent cells. Cells were incubated with AM841 (10^−6^ M) or vehicle in medium for 1 h and then treated with LPS (5 µg/ml) for 24–48 h at 37°C. Cell viability was assessed using Trypan blue staining method.

### Phagocytosis assay

Phagocytic activity after AM841 exposure was evaluated in colorimetric assay, as described previously [27]. Cells (5×10^5^ in 0.2 ml/well) were cultured on a 96-well plate and incubated for 30 min in cell medium containing neutral red stained zymosan (0.2×10^8^ particles/well). Cells were fixed with 100 µl formol-calcium solution (10% formalin, 2% sodium chloride, 1% calcium acetate) for 30 min and washed twice with PBS. Cell-associated stained zymosan was released by adding 0.1 ml of acidified alcohol (10% acetic acid, 40% ethanol in distilled water) to each well and incubation for 30 min at 37°C. Absorbance, measured at 550 nm using microplate reader (Thermo Fischer Labsystems Multiskan, Thermo Scientific, Ottawa, ON, Canada), was directly proportional to the amount of ingested neutral red stained zymosan.

### Neutral red uptake

The uptake of the cationic neutral red dye, which accumulates in cell lysosomes, was used to assess the integrity of the macrophage lysosomal system [28]. Cells (5×10^5^ in 0.2 ml/well) were seeded on a 96-well plate, incubated for 30 min at 37°C with 20 µl of 3% neutral red in PBS and rinsed twice with PBS. Neutral red was dissolved by 30 min incubation with 0.1 ml of 10% acetic acid and 40% ethanol solution added to each well. Absorbance, measured at 550 nm using microplate reader (Thermo Fischer Labsystems Multiskan, Thermo Scientific, Ottawa, ON, Canada), was directly proportional to the amount of neutral red.

### Statistics

Statistical analysis was performed using Prism 4.0 (GraphPad Software Inc., La Jolla, CA, USA). The data are expressed as means ± SEM. Student t-test or one-way ANOVA followed by Newman-Keuls post-hoc test were used for analysis. P values <0.05 were considered statistically significant.

### Drugs

All drugs and reagents, unless otherwise stated, were purchased from Sigma-Aldrich (Oakville, ON, Canada). DSS (MW 40,000) was purchased from MP Biomedicals (Solon, OH, USA). (−)-7′-isothiocyanato-11-hydroxy-1′, 1′-dimethylheptylhexahydrocannabinol (AM841) [29] was synthesized at the Center for Drug Discovery, Northeastern University Boston, MA (AM, GT, RT). 1-Naphthalenyl[4-(pentyloxy)-1-naphthalenyl]methanone (CB 13) was purchased from Tocris (Minneapolis, MN, USA).

## Results

### Cannabinoid receptor agonist AM841 protects against DSS-induced colitis in mice

**Figure 1:**
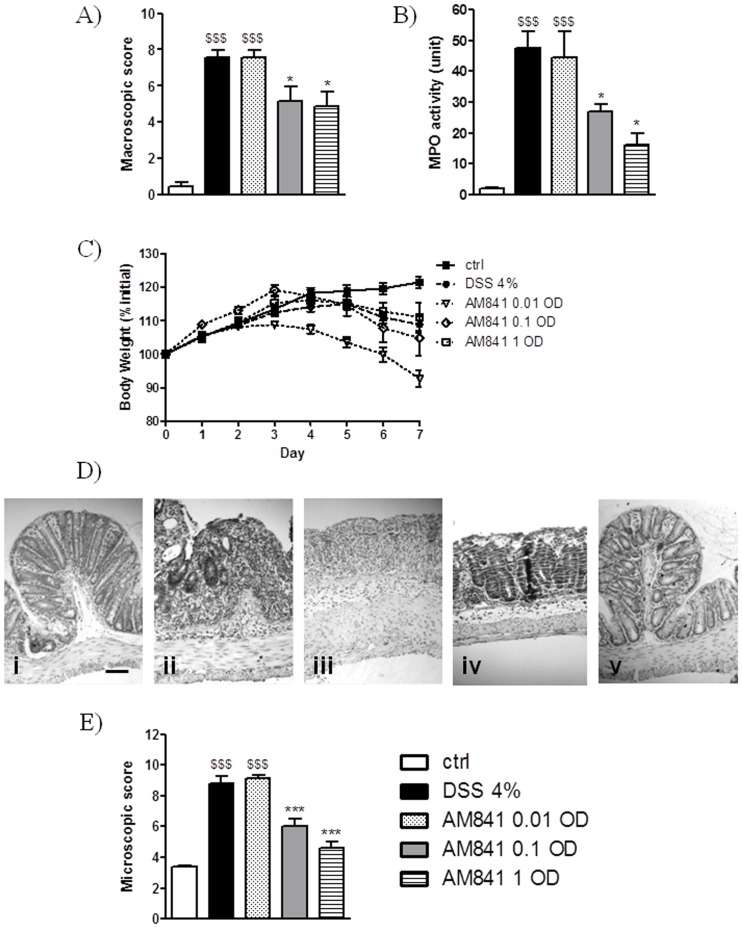
Protective effect of AM841 (0.01, 0.1 and 1 mg/kg, i.p.) injected once daily for 7 days on DSS-induced colitis in mice. Figure shows data for (A) macroscopic score, (B) MPO activity, (C) body weight, (D) representative micrographs for hematoxylin and eosin staining of colon wall sections (i, control; ii, DSS; iii, DSS+AM841 0.01 mg/kg; iv, DSS+AM841 0.1 mg/kg; v, DSS+AM841 1 mg/kg; scale bar = 100 µm) and (E) microscopic score. $$$p<0.001, as compared with control animals. *p<0.05, ***p<0.001, as compared with DSS-treated mice. Data represent means ± SEM, n = 6–8.

In order to evaluate the possible protective role of a CB receptor agonist on intestinal inflammation, we examined animals treated with DSS to induce colitis. Administration of DSS in drinking water resulted in development of intestinal inflammation, evidenced by a significant increase of macroscopic damage score (Fig. 1A) and MPO activity (Fig. 1B), as compared with animals receiving tap water. Treatment with the CB receptor agonist AM841 (0.01, 0.1 and 1 mg/kg, i.p.) injected 15 min before and once daily for 7 days following induction of colitis, significantly attenuated colonic inflammation (Fig. 1). The effect of AM841 on overall macroscopic damage score (7.58±0.40, 5.18±0.81, and 4.90±0.81 for AM841 at the dose of 0.01, 0.1 and 1 mg/kg, respectively vs. 7.59±0.41 for DSS-treated mice) and the MPO activity (44.4±8.5, 27.1±2.3, and 17.2±4.2 units for AM841 at the dose of 0.01, 0.1 and 1 mg/kg, respectively vs. 47.6±5.4 units for DSS-treated mice) was dose-dependent. However, the effect of AM841 at the lowest (0.01 mg/kg) and the highest (1 mg/kg) dose tested on colon weight and length, as well as stool score (data not shown) was almost equipotent.

The microscopic evaluation of colon sections stained with hematoxylin/eosin was in good agreement with the observation of the macroscopic parameters. AM841 dose-dependently attenuated inflammation by decreasing colonic tissue infiltration with immunocytes, improving mucosal and muscular architecture, and inhibiting ulceration (Fig. 1D). The microscopic damage score was significantly decreased by AM841 at the two higher doses tested (6.00±0.52 and 4.60±0.43 for 0.1 and 1 mg/kg, i.p., respectively vs. 8.83±0.48 for DSS-treated mice, Fig. 1E).

A potent anti-inflammatory effect of the CB agonist was also observed using a different type of dosing schedule. The i.p. administration of AM841 (0.1 mg/kg, i.p.) injected 15 min before and twice daily for 7 days following induction of colitis significantly decreased macroscopic (5.01±0.66 vs. 7.40±0.50 for DSS-treated mice) and microscopic colonic damage score (5.78±0.32 vs. 8.51±0.45 for DSS-treated mice). Interestingly, there was no additional beneficial effect of AM841 on intestinal inflammation associated with more frequent injections.

### Anti-inflammatory effect of AM841 is absent in CB receptor deficient mice

The effect of AM841 on colitis was next examined in CB_1_^-/-^, CB_2_^-/-^ and CB_1/2_^-/-^ mice. Since all CB receptor deficient mice used in the study had a C57Bl/6 background, preliminary assays were performed in order to establish the experimental conditions and DSS treatment. Since no significant differences between the parameters of inflammation were observed between DSS-treated CD1 and C57Bl/6 wild type, or wild type and CB knockout mice, therefore same DSS concentration and treatment schedule were employed.

**Figure 2:**
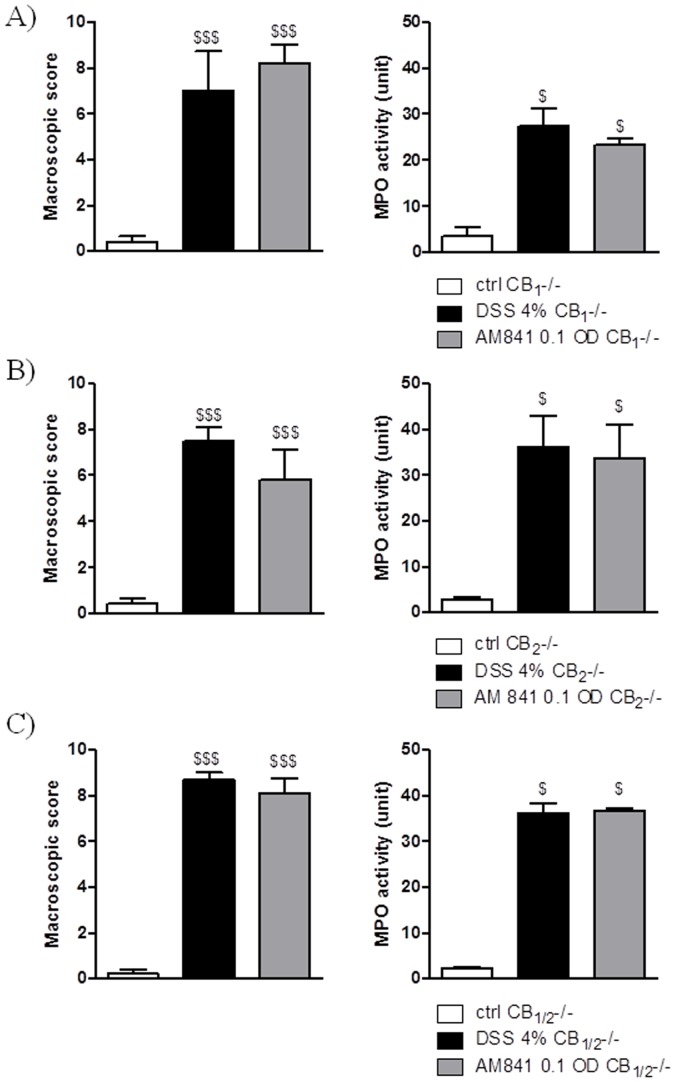
AM841 (0.1 mg/kg, i.p.) injected once daily for 7 days did not alter DSS-induced colitis in (A) CB1^-/-^, (B) CB2^-/-^ (C) and CB1/CB2^-/-^ mice. Figure shows data for macroscopic score and MPO activity. $p<0.05, $$$p<0.001, as compared with control animals. Data represent means ± SEM, n = 6–8.

The i.p. administration of AM841 at the effective dose in wild type animals (0.1 mg/kg, once daily) did not attenuate the DSS-induced colitis in CB_1_^-/-^ (Fig. 2A), CB_2_^-/-^ (Fig. 2B) or CB_1/2_^-/-^ mice (Fig. 2C). This lack of effect can be seen in the macroscopic damage score and MPO activity (Fig. 2), as well as microscopic colon damage score (data not shown).

### Cannabinoid receptor agonist AM841 heals colitis in mice

**Figure 3:**
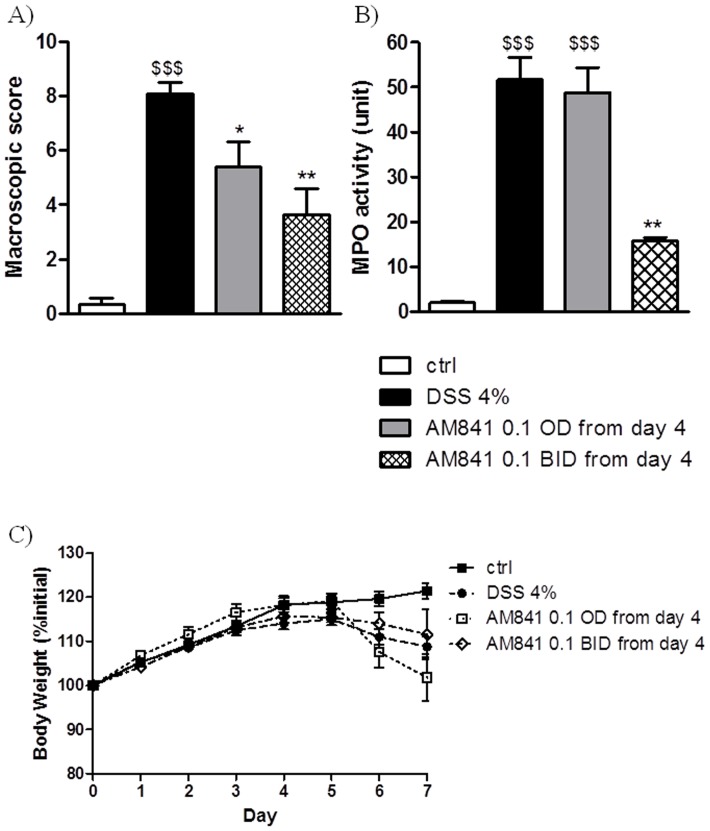
Therapeutic effect of AM841 (0.1 mg/kg, i.p.) injected once or twice daily from day 4 on DSS-induced colitis in mice. Figure shows data for (A) macroscopic score, (B) MPO activity and (C) body weight. $$p<0.01, $$$p<0.001, as compared with control animals. *p<0.05, **p<0.01, as compared with DSS-treated mice. Data represent means ± SEM, n = 6–8.

Next we examined the effect of CB receptor activation on intestinal inflammation in mice with clearly established colitis. At the beginning of the experiment animals were treated with DSS in drinking water and the development of intestinal colitis was monitored based on clinical scoring (changes in body weight and stool consistency, appearance of rectal bleeding). DSS-treated animals with significantly increased clinical scores were randomly assigned to groups receiving either vehicle or AM841 (0.1 mg/kg, i.p.) once or twice daily on days 4–7 after induction of colitis. Treatment with AM841 resulted in a significant attenuation of intestinal inflammation and a decrease of total macroscopic damage score (5.40±0.92 and 3.62±0.96 for AM841 injected once and twice daily, respectively vs. 8.09±0.43 for DSS-treated mice, Fig. 3).

### Cannabinoid receptor agonist AM841 protects against TNBS-induced colitis in mice

**Figure 4:**
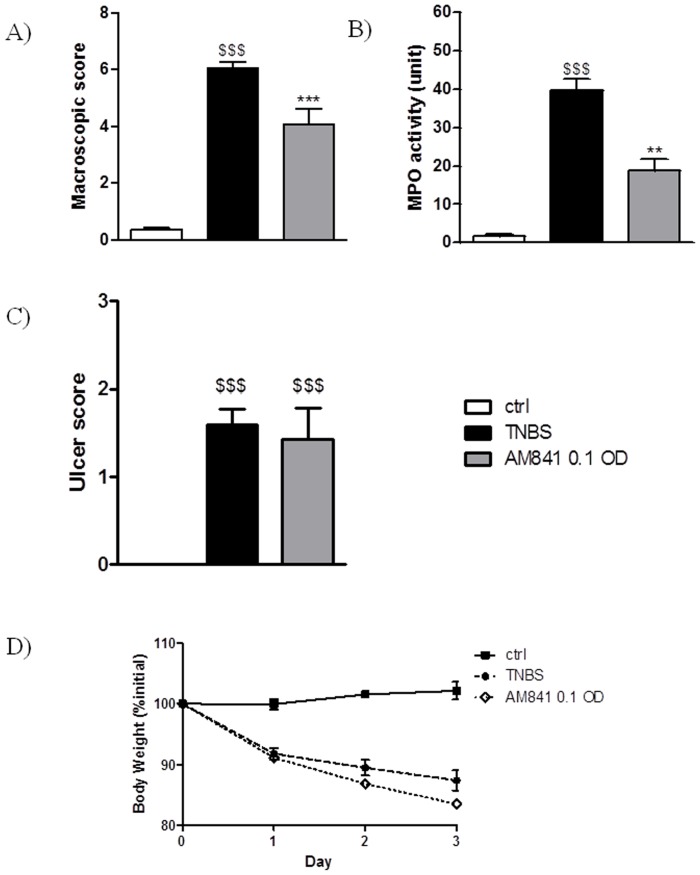
Protective effect of AM841 (0.1 mg/kg, i.p.) injected once daily for 3 days on TNBS-induced colitis in mice. Figure shows data for (A) macroscopic score, (B) MPO activity, (C) ulcer score and (D) body weight. $$$p<0.001, as compared with control animals. **p<0.01, ***p<0.001, as compared with TNBS-treated mice. Data represent means ± SEM, n = 6–8.

To confirm the protective effect of the CB receptor agonist AM841 against intestinal inflammation, the experiment was performed in TNBS-treated mice (Fig. 4). Intracolonic administration of vehicle (30% ethanol in saline) produced a minor inflammation, which was significantly different from the TNBS-induced colitis (macroscopic damage score 6.07±0.21, microscopic score 10.3±0.7, MPO activity 39.7±3.1 units in TNBS-treated mice, n = 5–6). AM841 (0.1 mg/kg, i.p., once daily 15 min before and for 3 days after induction of colitis) significantly attenuated intestinal inflammation, as shown by a reduction of macroscopic score (Fig. 4A) and MPO activity (Fig. 4B). No changes were seen in and ulcer score (Fig. 4C) or body weight (Fig. 4D) after treatment with AM841.

### Effect of AM841 on neutrophil migration, cell viability and phagocytic activity of splenocyte-derived macrophages

**Figure 5:**
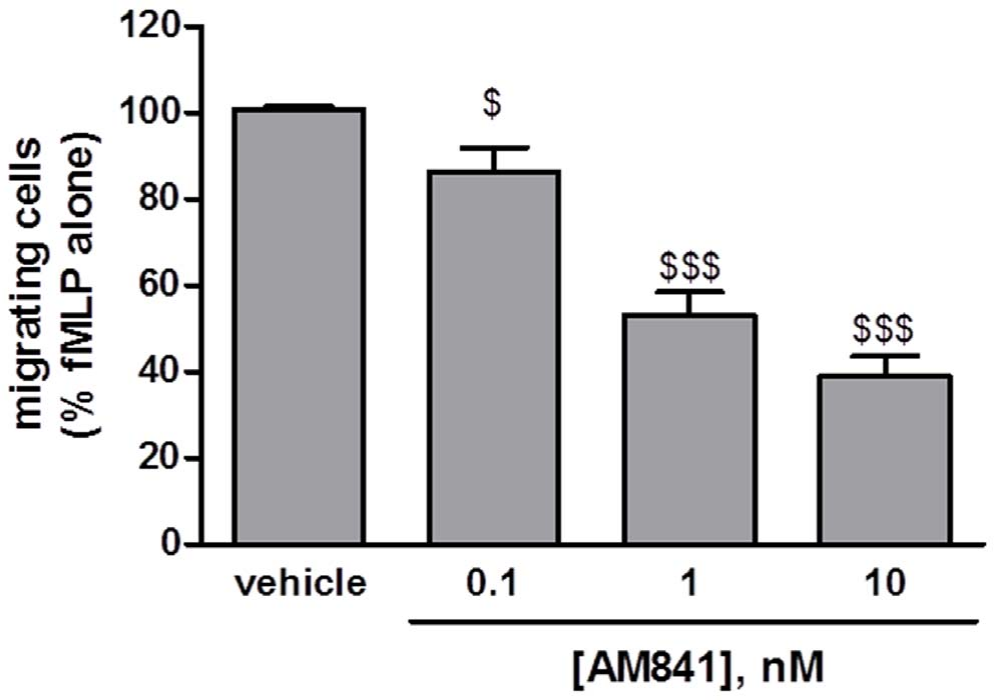
Chemotactic assay with murine neutrophils. AM841 (0.1–100 nM) inhibited neutrophil migration to 10 nM fMLP in a concentration-dependent manner. $p<0.05, $$$p<0.001, as compared with vehicle-treated controls. Data represent means ± SEM of 4 independent experiments performed in duplicate.

In order to characterize the molecular mechanism of anti-inflammatory action of AM841, we performed a chemotactic assay with neutrophils isolated from mouse bone marrow, using fMLP as chemoattractant. AM841 (0.1–10 nM) inhibited fMLP-stimulated neutrophil migration in a concentration-dependent manner (Fig. 5). The CB receptor agonist alone had no effect on spontaneous neutrophil migration.

To further investigate the possible action of AM841 in inflamed colonic tissues, macrophage-like cells were obtained from splenocytes isolated from control and DSS- or TNBS-treated mice. Incubation of macrophages isolated from control and DSS-treated animals with AM841 (10^-6^ M) for 48 h did not influence their viability as measured using a Trypan blue staining method (AM841 decreased the number of viable cells by 5.32±2.62 and 9.78±4.51% in control and DSS-treated animals, respectively). However, the number of cells obtained from mice with TNBS-induced colitis was significantly reduced by 52.94±6.97% after incubation with AM841.

AM841 (10^−6^ M) decreased phagocytic activity by 10.46±4.65 and 15.4±5.43% in DSS- and TNBS-treated animals, respectively. Neutral red uptake, which indicates the integrity of the macrophage lysosomal system, was inhibited by 15.38±7.37 and 14.28±9.46% in DSS- and TNBS-treated animals, respectively.

### Activation of peripheral cannabinoid receptors protects against DSS-induced colitis in mice

**Figure 6:**
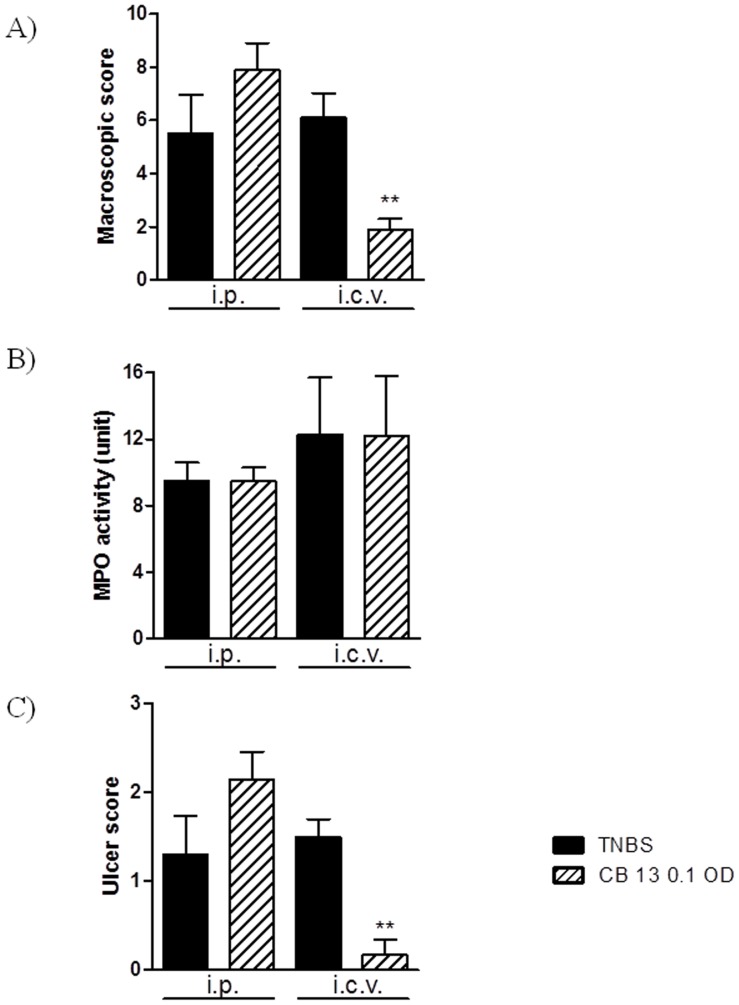
Protective effect of CB 13 after central (0.1 µg/animal, i.c.v, OD), but not peripheral (0.1 mg/kg, i.p., OD) administration for 3 days on TNBS-induced colitis in mice. Figure shows data for (A) macroscopic score, (B) MPO activity and (C) ulcer score. **p<0.01, as compared with TNBS-treated mice. Data represent means ± SEM, n = 6–8.

In an attempt to elucidate the role of peripheral CB receptors in the anti-inflammatory activity of cannabinoid agonists, the effect of a peripherally-restricted agonist CB 13 on TNBS-induced colitis was evaluated (Fig. 6). The i.p. administration of CB 13 (0.1 mg/kg) once daily 15 min before and for 3 days after induction of colitis did not influence the macroscopic damage score (Fig. 6A), MPO activity (Fig. 6B) or ulcer score (Fig. 6C). However, the peripherally-restricted CB agonist significantly attenuated macroscopic and ulcer scores (Fig. 6A and C, respectively) when injected centrally at the dose of 0.1 µg/animal.

## Discussion

The involvement of the ECS in the development of intestinal inflammation in animal models and human colitis has recently been suggested [17], [19]. The aim of the present study was to gain a deeper insight into the pharmacology of cannabinoids in colitis using high-affinity probes at CB receptors, and to see if the effects of CB receptor activation are centrally and/or peripherally-restricted. We observed a beneficial effect of CB receptor activation such that colitis was prevented or healed following treatment with AM841. We also showed that the presence of CB_1_ or CB_2_ receptors is sufficient to confer the anti-inflammatory effects of cannabinoids in these experimental models of colitis. Finally, we demonstrated that for full protection, central and peripheral CB receptors are required.

AM841 is a novel cannabinergic ligand that was designed to interact with specific amino acid residues at or immediately adjacent to the CB_1_ and/or CB_2_ receptor-binding pocket [23], [30]. AM841 has been very effective in selective binding to CB_1_ receptors in rat brain synaptosomes [23] and hCB_2_ in transfected HEK293 cell line [30]. The remarkable specificity and potency at CB receptors make AM841 as useful tool in studying the involvement of cannabinoids in physiological processes, both *in vitro* and *in vivo*. In addition, unlike many cannabinoids including the endogenous CB ligand anandamide, AM841 does not activate non-CB receptor targets, such as the vanilloid receptor TRPV1 [31]. CB 13 on the other hand is a recently developed, potent orally bioavailable human CB1/CB2 dual agonist.

In our study, AM841, but not CB 13 was effective after peripheral administration, suggesting that a central component is important in the anti-inflammatory actions of CB agonists. These data are consistent with the observations made by Cluny et al., who showed that the peripherally restricted CB agonist SAB378 was ineffective at reducing colitis [32]. The most striking observation here was that the anti-inflammatory effect of CB 13 was revealed after central administration, as demonstrated by a significant decrease of macroscopic damage and ulcer score. The activation of centrally located CB receptors seems thus crucial for the anti-inflammatory action of cannabinoids and this observation may have a tremendous impact on further development of this class of compounds for potential clinical application in colonic inflammation treatment.

An important contribution to our understanding of the CB receptor pharmacology is the absence of an anti-inflammatory effect of AM841 in CB_1_^-/-^, CB_2_^-/-^ and CB_1/2_^-/-^ mice. Contrary to what we expected AM841, a CB agonist with a considerable preference to CB_1_ over CB_2_ receptors, did not attenuate colitis in animals lacking the CB_2_ receptor, which confirms our earlier observations [19]. Our results indicate that the expression of both CB receptors is required for the ECS to protect against colitis and that the drugs non-selectively targeting CB_1_ and CB_2_ could be more efficient anti-inflammatory therapeutics. Further studies are thus required to explain this phenomenon.

The protective effect of cannabinoids on colitis and the involvement of the CB receptors in mediation of their anti-inflammatory effects have been demonstrated [15]–[19]. In our study, as expected, both CB agonists produced a significant protective effect on DSS- and TNBS-induced colitis. A key finding of our study is, in addition to well-documented protective effects, the ability of CB receptor agonists to heal the intestinal inflammation at an advanced stage. Previously published data indicated a possible protective effect of cannabinoids in mustard oil -induced colitis, which is regarded as a model for neurogenic colonic inflammation [16]. Kimball et al. [16] showed that the CB_1_ receptor agonist attenuated mustard oil-colitis by prophylactic and therapeutic dosing regimen, whereas the CB_2_ agonist was more effective in late administration. These results, along with our observations, imply the crucial role of ECS in the development of colitis and suggest a potential application of CB receptor agonists in the treatment of colonic inflammation even at later time points. Interestingly, MPO activity, which is an indicator of neutrophil infiltration, but not macroscopic or microscopic scores were affected by a dosing schedule of AM841 applied in a therapeutic manner in DSS-treated mice. This observation suggests that the activation of the immune system is crucial for therapeutic effects of cannabinoids and suggested that the CB_2_ receptors, which are predominantly expressed on neutrophils, may be important mediators of their anti-inflammatory action.

The influence of AM841 on migratory properties of neutrophils *in vivo* was subsequently investigated and confirmed in the chemotactic assay *in vitro*. AM841 concentration-dependently inhibited chemoattractant-stimulated migration of neutrophils. This observation, which is in line with previous reports showing the ability of anandamide, 2-AG and synthetic cannabinoids to inhibit neutrophil migration in response to fMLP [33] and other chemoattractants [34], [35] bears great therapeutic potential. Infiltration of leukocytes, primarily neutrophils, and their inappropriate activation can cause local inflammation and increase the release of pro-inflammatory cytokines, leading to a number of autoimmune, inflammatory, and neoplastic disorders (for review see: [36]). Inhibition of neutrophil chemotactic activity might thus be an important feature of the anti-inflammatory action of cannabinoids in colitis. However, although the presence of the CB receptors on neutrophils was described [10], [13], it was also suggested that the action of cannabinoids could be mediated by CB_1_/CB_2_-independent mechanisms [33]. Therefore further studies are required to elucidate the role of CB receptors in neutrophil-related effects.

Studies *in vitro* have shown that cannabinoids reduced proliferative responses of mouse splenic T and B cells [37]–[39], cytotoxic T cell activity [40], and antibody synthesis [41]. In an attempt to further characterize the molecular mechanisms underlying the anti-inflammatory action of cannabinoids, we examined the effect of AM841 on proliferative response of splenocyte-derived macrophages. Macrophages are an important element of immune defence systems, as they engulf and destroy the antigens upon activation of innate immunity, as well as present processed antigens to immunologically naive lymphocytes in an early recognition phase of acquired immunity [42] and were thus examined in our study. The expression of CB1 and CB2 receptors on macrophages has also been demonstrated [18], [43]. Interestingly, the CB receptor agonist AM841did not affect the proliferation of macrophages obtained from DSS-treated mice, but significantly decreased the viability of cells from the TNBS-treated animals. Furthermore, the incubation of splenocyte-derived macrophages with AM841 did not affect their phagocytic activity. This is in good agreement with a study by Mann et al. [44], who observed that the exposure to marijuana smoke did not alter the phagocytic capacity, but caused metabolic and morphological changes in alveolar macrophages from humans and rats, but contradicts other studies showing that macrophage spreading, phagocytosis and cell metabolism may be modulated through CB receptor-dependent mechanisms [45]–[48]. The clinical relevance of our findings remains unclear, but suggests the immunomodulatory potential of macrophages, which may become a future target for cannabinoids in the treatment of intestinal inflammation.

## Conclusion

In conclusion, in the present study we showed that the CB receptor agonists AM841 and CB 13 displayed protective and therapeutic effects on colitis in mice. Most importantly, we demonstrated that the anti-inflammatory action of the cannabinoids was mediated through CB_1_ and CB_2_ receptors localized centrally and possibly to a lesser extent - peripherally. To the best of our knowledge, this is the first evidence showing the involvement of central CB receptors in development and treatment of colitis. These results are crucial for our understanding of the pharmacology of CB ligands in GI inflammation and may have potential applications in the development of future treatment strategies for IBD in humans.
